# Flavagline synthetic derivative induces senescence in glioblastoma cancer cells without being toxic to healthy astrocytes

**DOI:** 10.1038/s41598-020-70820-6

**Published:** 2020-08-13

**Authors:** Ezeddine Harmouch, Joseph Seitlinger, Hassan Chaddad, Geneviève Ubeaud-Sequier, Jochen Barths, Sani Saidu, Laurent Désaubry, Stéphanie Grandemange, Thierry Massfelder, Guy Fuhrmann, Florence Fioretti, Monique Dontenwill, Nadia Benkirane-Jessel, Ysia Idoux-Gillet

**Affiliations:** 1 INSERM (French National Institute of Health and Medical Research), UMR 1260, Regenerative Nanomedicine (RNM), FMTS, 11 Rue Humann, 67000 Strasbourg, France; 2 Faculté de Chirurgie Dentaire, Université de Strasbourg, 67000 Strasbourg, France; 3 Hôpitaux Universitaire de Strasbourg (HUS), 67000 Strasbourg, France; 4 Core Facility for Flow Cytometry, Cell Sorting and EliSpot, UMR 1110, INSERM, Strasbourg, France; 5 CNRS UMR 7021, Laboratoire de Bioimagerie et Pathologies, Faculté de Pharmacie, Strasbourg, France; 6 Laboratory of Cardio-Oncology and Medicinal Chemistry (FRE 2033), CNRS, Institut Le Bel, Strasbourg, France; 7 Sino-French Joint Lab of Food Nutrition/Safety and Medicinal Chemistry, College of Biotechnology, Tianjin University of Science and Technology, Tianjin, China; 8 CNRS, UMR 7039 CRAN, Université de Lorraine, Campus Sciences, 30 bvd des Aiguillettes, 54505 Vandoeuvre les Nancy Cedex, France

**Keywords:** Cancer, Cell biology, Health care, Oncology

## Abstract

Glioblastoma (GBM) is one of the most aggressive types of cancer, which begins within the brain. It is the most invasive type of glioma developed from astrocytes. Until today, Temozolomide (TMZ) is the only standard chemotherapy for patients with GBM. Even though chemotherapy extends the survival of patients, there are many undesirable side effects, and most cases show resistance to TMZ. FL3 is a synthetic flavagline which displays potent anticancer activities, and is known to inhibit cell proliferation, by provoking cell cycle arrest, and leads to apoptosis in a lot of cancer cell lines. However, the effect of FL3 in glioblastoma cancer cells has not yet been examined. Hypoxia is a major problem for patients with GBM, resulting in tumor resistance and aggressiveness. In this study, we explore the effect of FL3 in glioblastoma cells under normoxia and hypoxia conditions. Our results clearly indicate that this synthetic flavagline inhibits cell proliferation and induced senescence in glioblastoma cells cultured under both conditions. In addition, FL3 treatment had no effect on human brain astrocytes. These findings support the notion that the FL3 molecule could be used in combination with other chemotherapeutic agents or other therapies in glioblastoma treatments.

## Introduction

Flavaglines are natural products isolated from *Aglaia* genus plants possessing unique anticancer properties. One synthetic flavagline, called FL3, is known for its anticancer effects without being toxic to healthy cells^1,2^. Flavaglines were isolated for the first time in 1982 based on their strong anti-leukemic activity^3^. Cytotoxic effects of flavaglines has been reported in a lot of cancer cell lines, such as lung, breast, and colon cancer^4^, leading to the inhibition of proliferation and thus inducing cell cycle arrest or apoptosis in tumor cells. Different mechanisms by which FL3 targets cancer cells were reported in the literature. It was shown that in urothelial carcinoma cells, FL3 can directly binds to Prohibitin 1 (PHB) preventing its phosphorylation by Akt, leading to a decrease of PHB in mitochondria, which causes a mitochondria-related apoptosis and cell cycle inhibition^5,6^. PHB is an ubiquitous and evolutionarily conserved protein expressed in different cellular compartments including the nucleus, cytoplasm and mitochondria^7^, it is involved in diverse biological processes such as cell proliferation, resistance to apoptosis, maintenance and integrity of mitochondria^7,8^. Also, FL3 was shown to selectively kill cancer stem-like cells through the p38 mitogen-activated protein kinase (MAPK)-dependent caspase-3-dependent pro-apoptotic pathway, without being toxic to normal stem-like cells^9^. Recently, it has been reported that mitophagy, a process that selectively removes damage or unwanted mitochondria in order to maintain normal cellular physiology, was inhibited by FL3 contributing to the blockage of cancer cell growth^10^.

In this study, we used four different glioblastoma cancer cell lines: U87-MG (both TMZ-sensitive and TMZ-resistant cells), U373-MG (p53-mutated) and LN443 (p53 WT) malignant glioma cells. Glioblastoma (GBM) is the most common type of primary brain tumor^11,12^, with a rapid growth and aggressive properties leading to an overall survival average of 14 to 18 months^13,14^. This tumor can be found anywhere in the brain and is predominantly composed of abnormal astrocytes but also a mix of different cell types. GBM often benefits from the selective conditions present in the tumor microenvironment. Generation of a hypoxic environment and activation of its main effector, hypoxia-inducible factor-1 (HIF-1), are common features of advanced GBM cancer stages^15^. Low tumor oxygenation promotes tumor cells invasion into the healthy brain tissue^16–18^. Hypoxia is therefore a major problem for patients with GBM, resulting in tumor resistance and aggressiveness. Due to the cellular heterogeneity inside this tumor, the first step of GBM treatment is a surgical removal of the tumor mass. Then radiation therapy and chemotherapy (based on the use of Temozolomide: DNA alkylating agent and the standard chemotherapeutic drug for GBM) are performed in order to kill remaining tumor cells. EGFR amplifications occur in more than 50% of glioblastomas^19^. Drugs targeting the constitutively active form of RTKs (ex: EGFR) and its downstream MAPK/PI3K signalling pathways, are particularly studied as glioblastoma targeted therapies^20^. Afatinib is a well-known drug capable of crossing the blood brain barrier BBB^21^ and directly target the EGFR thus limiting the proliferation and invasion of glioblastoma cancer cells. But due to the limited efficacy of this treatment, a new anticancer model has been established combining the Afatinib drug with the TMZ. This new system of anticancer therapy combination significantly reduces the glioblastoma tumor growth both in vitro and in pre-clinical mouse models^22^. All of the new targeted therapies (for example against EGFR) failed in clinical trials and GBM remain a challenge for oncologists.

These conventional therapies target mostly high proliferative and well-oxygenated cells. The challenge consists in targeting hypoxic cancer cells, known to be more aggressive and resistant to anticancer treatments^23^.

As FL3 displays potent anticancer activities and is known to inhibit cell proliferation in many cancer cells, we examined its effect on different glioblastoma cell lines and compared normoxic and hypoxic conditions. Also we studied whether FL3 could be toxic for primary human brain astrocytes.

## Results

### FL3 blocks U87-MG cell proliferation at G2 phase, without inducing apoptosis

**Figure 1:**
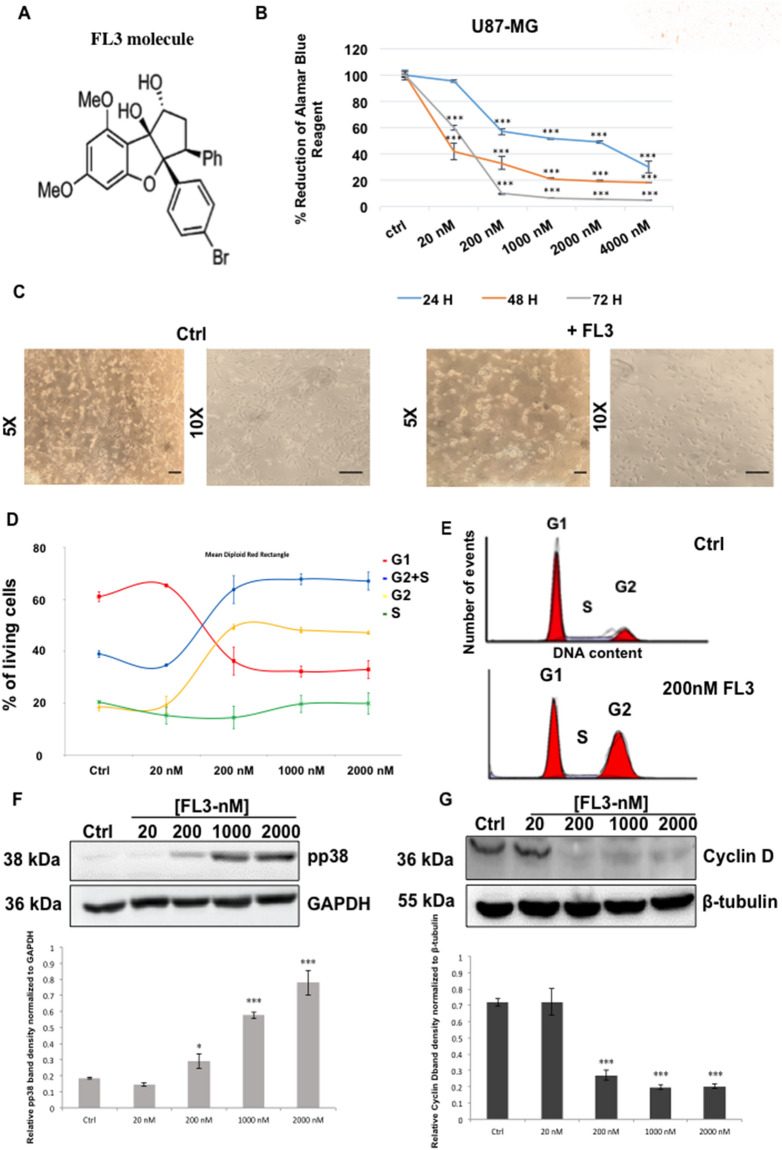
Effect of FL3 on U87-MG glioblastoma cancer cells metabolic activity and proliferation. (**A**) Chemical structure of FL3. (**B**) Analysis of cell viability with the AlamarBlue assay at different time points in U87-MG control cells and U87-MG treated cells with different concentrations of FL3. (**C**) Representative images of control cells and U87-MG cells treated with 200 nM FL3. The scale bar represents 200 μm. (**D**) Distribution of cells in each cell cycle phase in control and treated conditions. (**E**) Representative histogram of cell cycle analysis of U87-MG cells treated with 200 nM FL3. (**F**) Western Blot and quantification of pp38 protein expression in control cells and U87-MG cells treated with different concentrations of FL3. (**G**) Western Blot and quantification of Cyclin D protein expression in control cells and U87-MG cells treated with different concentrations of FL3. Quantification of mean ± SEM from 3 independent experiments, *P < 0.05, **P < 0.01, ***P < 0.001 from control. n = 3 for each concentration.

**Figure 2:**
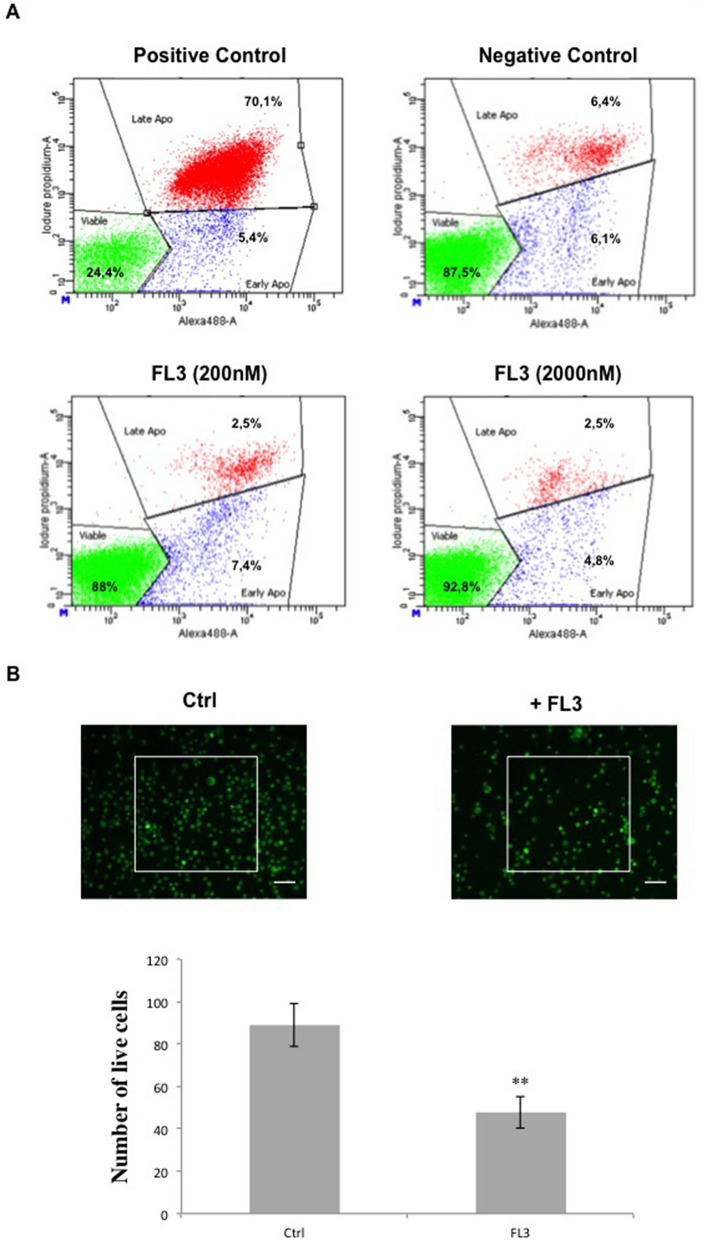
Analysis of the apoptosis in glioblastoma cancer cells. (**A**) Flow cytometry analysis of Annexin V and PI staining in U87-MG cells treated with 200 and 2000 nM FL3 and compared to positive and negative controls. We used an NF-κB inhibitor as positive control. Results are obtained from 3 independent experiments. (**B**) Live/dead assay on U87-MG cells treated or not with 200 nM FL3 and quantification of live cells in each condition. The scale bar represents 20 μm. Cells within white boxes were counted at each time. Quantification of mean ± SEM from 3 independent experiments, **P < 0.01 from control. n = 3 for each condition.

First, cell viability was analysed by the Alamar Blue assay at different time points of treatment and using different concentrations of FL3 (chemical structure: Fig. 1A) on U87-MG cells. Our results showed no significant decrease of the Alamar Blue reduction percentage for 20 nM of FL3 after 24 h treatment, but around 50% loss of viability after 48 h and 72 h of treatment (Fig. 1B). A drastic decrease of metabolic activity appeared with a concentration of 200 nM for 24 h of treatment and continued to decrease for 48 h and 72 h. Beyond 200 nM, the loss of cell viability seemed to reach saturation for all treatment time points. To establish a good ratio between the treatment time and the concentration of FL3, we chose to treat the cells for 48 h with 200 nM of FL3. Therefore, the decrease in metabolic activity, cell density associated to microscopic observation (Fig. 1B, C) indicates that FL3 is cytostatic or cytotoxic in U87-MG cells. Cell cycle analysis showed an increased number of cells in the G2 phase after FL3 treatment in a dose dependant manner (Fig. 1D, E). These results were supported by the upregulation of phospho-p38 MAPK protein (Mitogen-activated protein kinases) and the downregulation of Cyclin D expression, known to be involved in the G2 cell cycle arrest (Fig. 1F and G). Interestingly, flow cytometry using Annexin V and Propidium Iodide apoptosis assays revealed that FL3 treatment does not induce cell death in U87-MG cells (Fig. 2A). Moreover, no cleaved-PARP protein has been detected in these treated cells (Fig. 6C). Also live/dead assays showed less cells in treated condition with FL3 compared to the control, and most importantly there were no cells stained in red, corresponding to dead cells, neither in treated nor in control conditions (Fig. 2B).

### FL3 induces senescence in glioblastoma cancer cells

**Figure 3:**
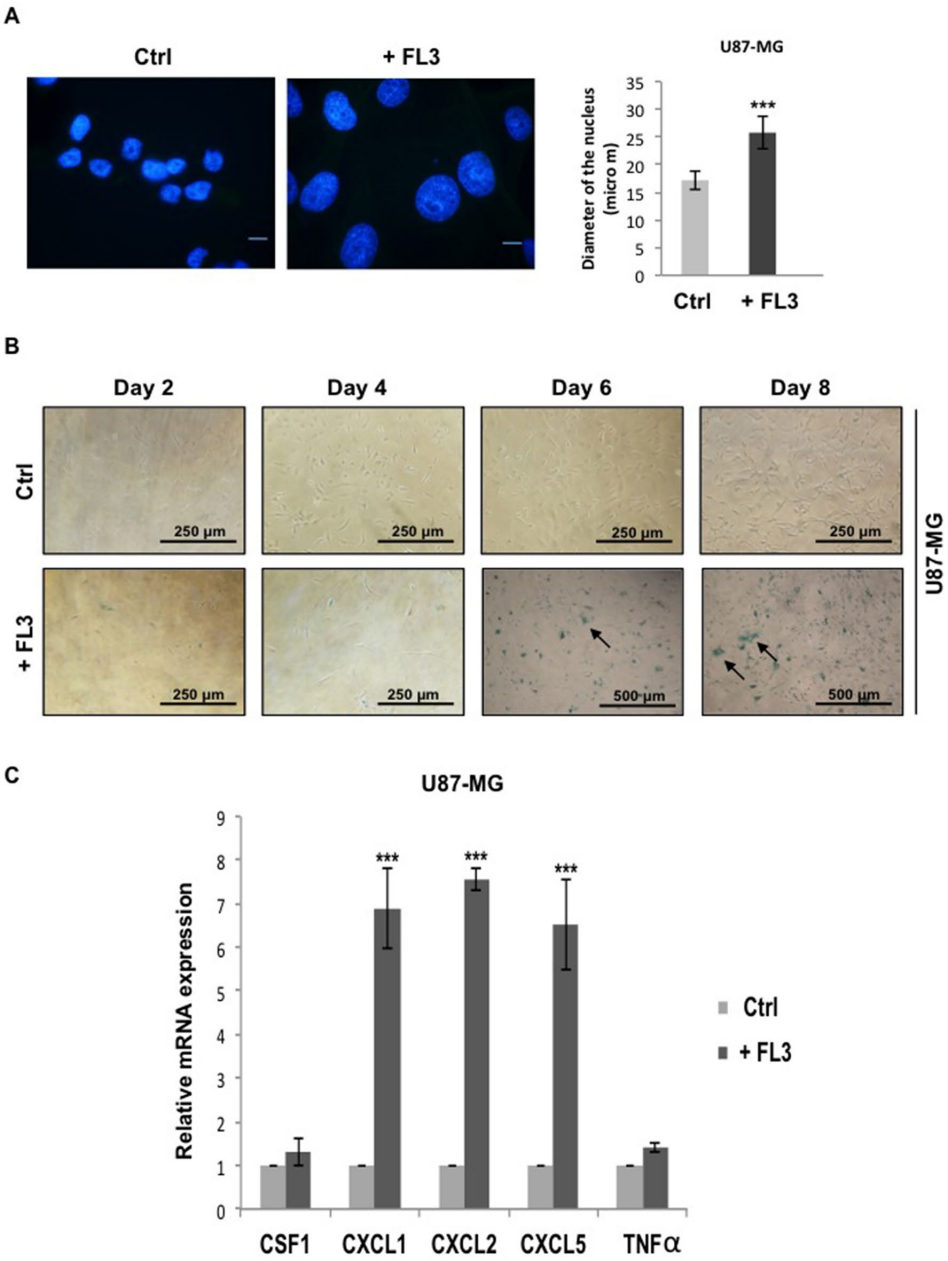
FL3 induced cellular senescence in U373 and LN443 glioblastoma cancer cells. (**A**) Observation and quantification of the nuclear shape and size in U87-MG control and treated cells with 200 nM FL3. 50 nuclei were measured for each condition. The scale bar represents 15 μm. Quantification of mean ± SEM from 3 independent experiments, ***P < 0.001 from control. (**B**) Representative senescence-associated β-galactosidase (SA β-gal) staining (blue) of control cells and U87-MG cells treated with 200 nM FL3, respectively. Pictures were taken at 2, 4, 6 and 8 days post-FL3 treatment. Black arrows indicate U87-MG positive cells. The scale bar represents: 250 and 500 µm. (**C**) Relative mRNA expression levels of SASP in control and treated U87-MG cells 6 days post treatment with 200 nM FL3. Quantification of mean ± SEM from 3 independent experiments, ***P < 0.001 from control.

As no sign of apoptosis was seen, we aimed to further understand the effect of FL3 on these U87-MG cancer cells. First, by using Hoechst staining, we observed that cells treated with FL3 presented much bigger nuclei compared to the control cells as shown in Fig. 3A, with an average nucleus size of 17 µm for untreated cells and 25 µm for FL3 treated cells (Fig. 3A). Since glioblastoma cells exhibit increased nuclear size with senescence^24^, we investigated whether FL3 induces senescence in U87-MG cells. Thus, senescence-associated β-Galactosidase assays have been performed on FL3 treated U87-MG cells, which were maintained for 2, 4, 6 and 8 days post-FL3 treatment. We observed that the majority of cells were positively stained in blue, 6 and 8 days post-FL3 treatment (Figs. 3B, 6E). These results were correlated with the remarkable increase in the transcript levels of the negative cell cycle progression regulators p16, p19 and p21, 6 days post-FL3 treatment (Fig. 6F). Notably, transcript levels of some members of the senescence associated secretory phenotype (SASP; e.g. CXCL1, CXCL2, and CXCL5) were also markedly increased 6 days post-FL3 treatment compared to the control (Fig. 3C). Thus, FL3 eventually induced senescence in U87-MG cells.

**Figure 4:**
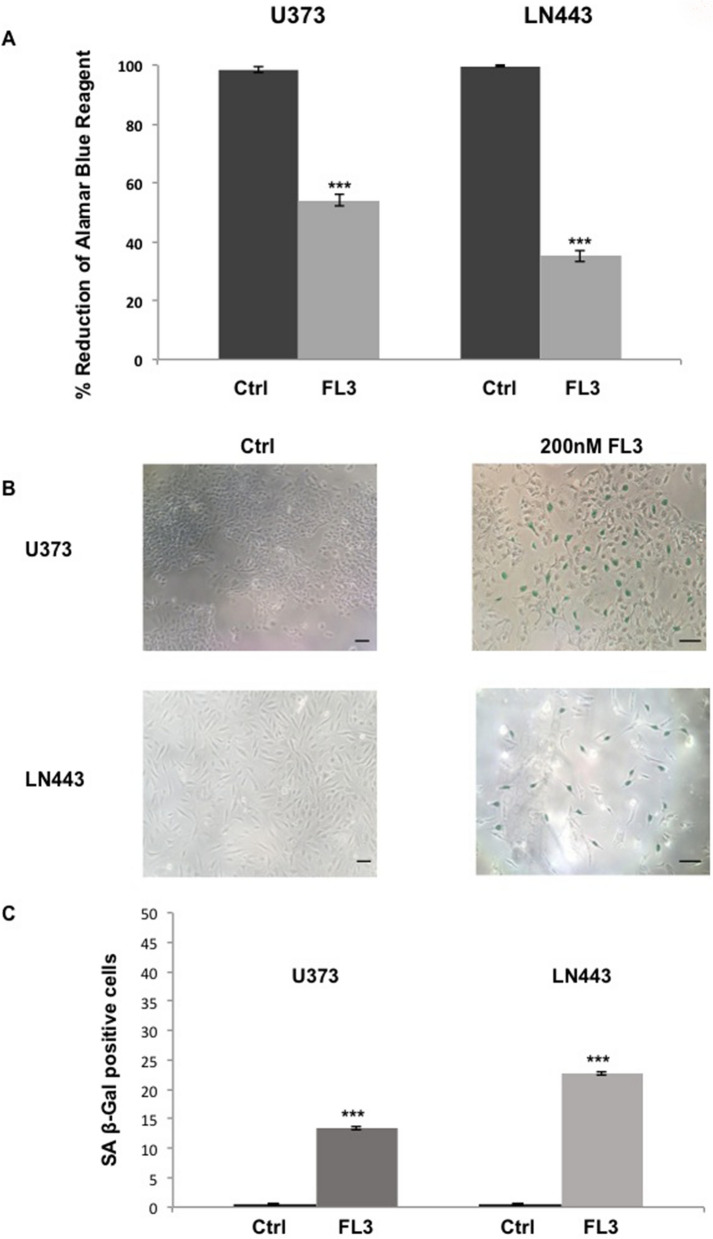
FL3 induced cellular senescence in U373-MG and LN443 glioblastoma cancer cells. (**A**) Analysis of cell viability with the AlamarBlue assay in U373-MG and LN443 control cells and treated cells with 200 nM of FL3, 48 h after the treatment. Quantification mean ± SEM from 3 independent experiments, ***P < 0.001 from control. (**B**) Representative senescence-associated β-galactosidase (SA β-gal) staining (blue) of U373 and LN443 control cells and treated cells with 200 nM FL3, 6 days after the treatment. Scale bar represents 150 μm. (**C**) Quantification of SA β-gal positive cells of U373 and LN443 control cells and treated cells with 200 nM of FL3. 50 cells were analysed for each condition. Results for mean ± SEM from 3 independent experiments, ***P < 0.001 from control.

In order to confirm the results obtained from U87-MG cells, we tested the effect of FL3 on two other glioblastoma cancer cell lines: U373-MG and LN443 cancer cells. We saw that the metabolic activity of both U373-MG and LN443 cells treated with 200 nM of FL3 was reduced compared to the control (for U373 cells, 45% decrease compared to control, and for LN443 cells, 65% decrease compared to control) (Fig. 4A). Most importantly, both U373-MG and LN443 treated cells with FL3 showed a blue positive staining for the senescence-associated β-Galactosidase assay, 6 days after the treatment (Fig. 4B, C). Thus, FL3 induced also senescence in U373-MG and LN443 glioblastoma cells.

### Hypoxic conditions do not interfere with FL3 effect

**Figure 5:**
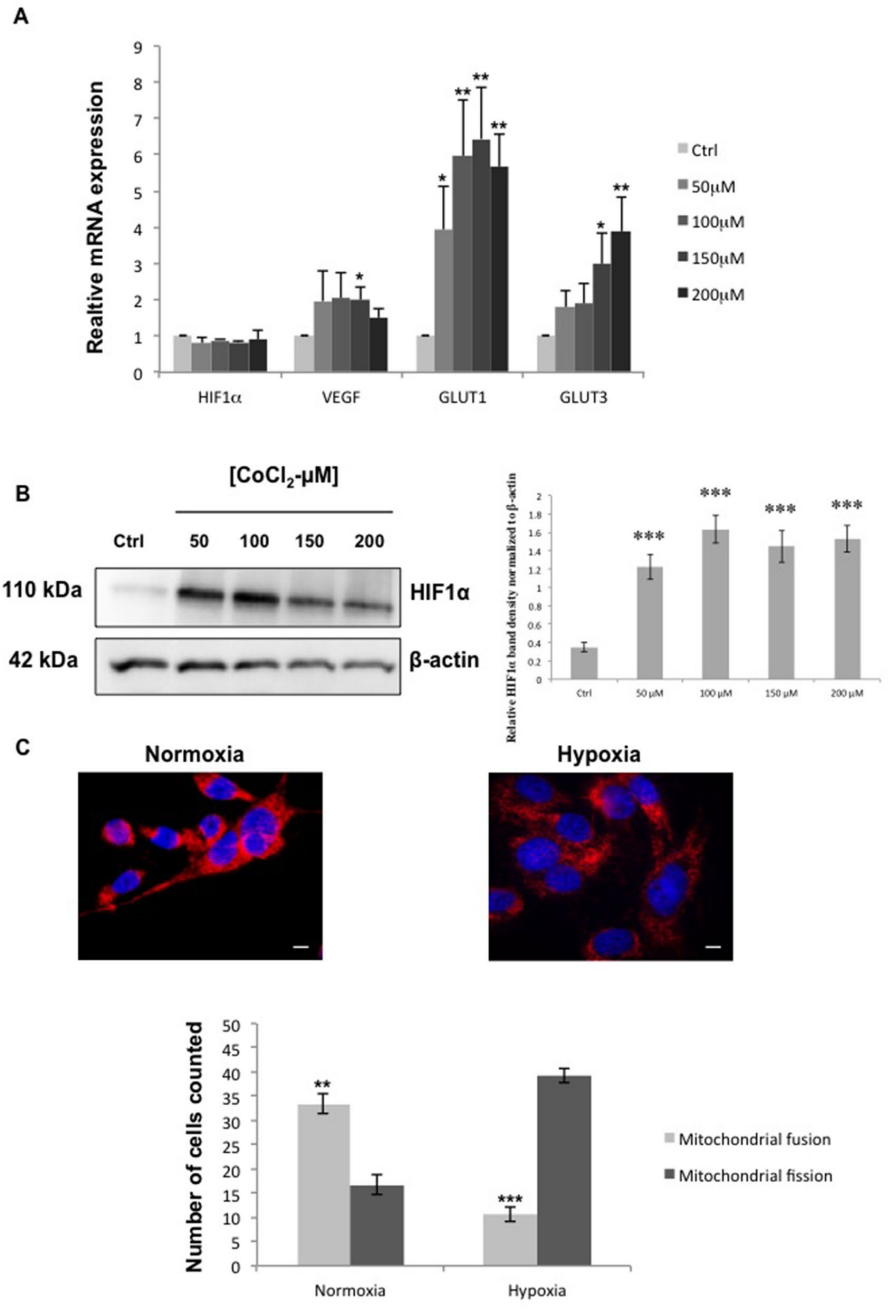
Characterization of the hypoxic model for glioblastoma cancer cells. (**A**) Relative mRNA expression levels of hypoxia related genes in control cells and U87-MG cells treated with different concentrations of CoCl_2_ after 24 h of treatment. Quantification of mean ± SEM from 3 independent experiments, *P < 0.05, **P < 0.01 from control. (**B**) Western Blot and quantification of HIF1α protein expression in control cells and U87-MG cells treated with different concentrations of CoCl_2_ after 24 h of treatment. Quantification of mean ± SEM from 3 independent experiments, ***P < 0.001 from control. (**C**) Immunofluorescence detection of Cytochrome C (red), in U87-MG cells cultured under normoxia and hypoxia. Blue staining shows the cell nucleus. The scale bar represents 50 µm. ImageJ (1.52a Version, https://imagej.nih.gov/ij/) was used to do our images. Quantification of fusioned and fissioned mitochondria inside U87-MG cells cultured under normoxia and hypoxia. 50 cells were analysed for each condition. Quantification of mean ± SEM from 3 independent experiments, **P < 0.01, ***P < 0.001 from control.

To induce hypoxia in U87-MG cells, we added cobalt chloride (CoCl2) to the regular culture medium. This agent is known to mimic hypoxia via inhibition of the HIF1α protein degradation in these cells^25,26^. Different CoCl_2_ concentrations were tested, and as expected, our results showed similar transcript levels of HIF1α in controls and CoCl2 treated U87-MG (Fig. 5A), whereas protein levels of HIF1α were increased in U87-MG cells treated with 50 to 200 µM of CoCl_2_ compared to control ones (Fig. 5B). Moreover, HIF1α target genes such as GLUT1, GLUT3, and VEGF were upregulated inside U87-MG cells treated with all concentrations of CoCl_2_ compared to control cells. In view of our results we chose to use 150 µM of CoCl_2_ in our experiments. To confirm that glioblastoma cells are in hypoxia we evaluated the mitochondrial state. Fluorescence staining with an anti-Cytochrome C antibody showed that CoCl_2_ treated cells presented mitochondrial fission compared to untreated cells exhibiting mitochondrial fusion (Fig. 5C). In 50 counted cells, the ratio between mitochondrial fusion and mitochondrial fission was reversed in U87-MG cells cultured in normoxia and in hypoxia, showing that U87-MG cells treated with 150 µM of CoCl_2_ were under hypoxic conditions.

**Figure 6:**
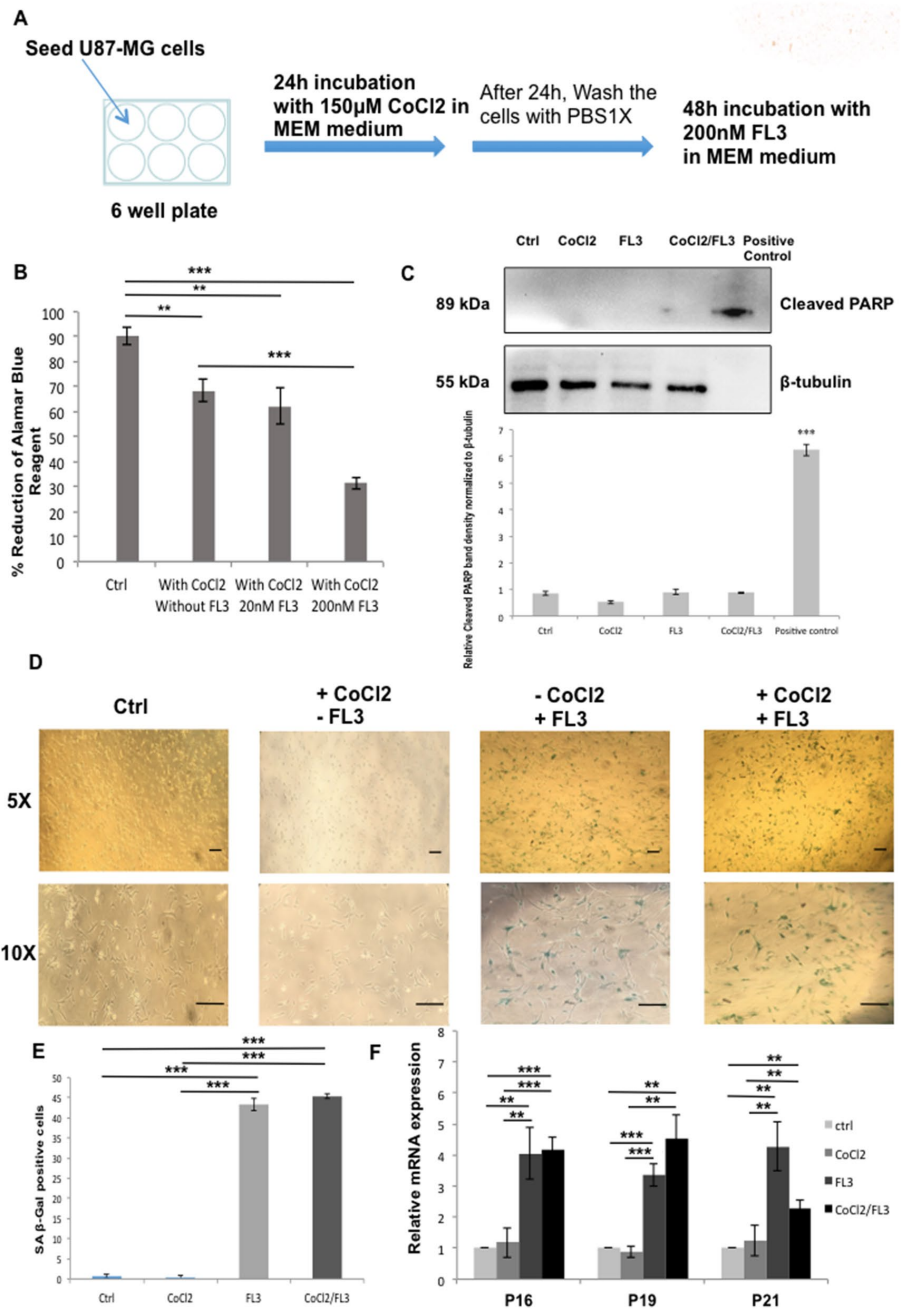
Effect of FL3 on hypoxic glioblastoma cancer cells model. (**A**) Representation of the experimental protocol used for the incubation of U87-MG cells with CoCl_2_ and FL3. (**B**) Analysis of cell viability with the AlamarBlue assay in control cells and U87-MG cells treated with two concentrations of FL3 (20 nM and 200 nM), under hypoxia (CoCl_2_). Quantification mean ± SEM from 3 independent experiments, **P < 0.01, ***P < 0.001 from control, and from +CoCl_2_. (**C**) Western Blot and quantification of cleaved PARP protein levels in control cells and U87-MG cells treated with 200 nM FL3 under normoxia and hypoxia (CoCl_2_). Cleaved PARP protein was used as positive control. Quantification mean ± SEM from 3 independent experiments, ***P < 0.001 from control. (**D**) Representative senescence associated β-galactosidase staining (blue) of control cells, treated cells with CoCl_2_ and U87-MG cells treated with 200 nM FL3, under normoxia and hypoxia (CoCl_2_), 6 days after the FL3 treatment. The scale bar represents 200 μm. (**E**) Quantification of SA β-gal positive cells of control cells, treated cells with CoCl2 and treated U87-MG cells with 200 nM FL3, under normoxia and hypoxia (CoCl_2_). 50 cells were analysed for each condition. Results for mean ± SEM from 3 independent experiments, ***P < 0.001 from control, and from +CoCl_2_. (**F**) Relative mRNA expression levels of cell cycle inhibitors in control cells and U87-MG cells treated with 200 nM FL3 6 days post FL3 treatment, under normoxia and hypoxia (CoCl_2_). Quantification of mean ± SEM from 3 independent experiments, **P < 0.01, ***P < 0.001 from control, and from +CoCl_2_.

To examine the effect of FL3 on U87-MG cells cultured under hypoxic conditions, cells were treated for 24 h with CoCl_2_ and then incubated with FL3 for 48 h (Fig. 6A), before performing Alamar Blue assays (Fig. 6B). A limited decrease of the metabolic activity of U87-MG cells under hypoxia compared to normoxia was observed. There was no significant difference when we added 20 nM of FL3. However, we observed a drastic decrease in the metabolic activity of U87-MG cells treated with CoCl_2_ and incubated with 200 nM FL3. These cells did not show any sign of apoptosis, there was no expression of the cleaved PARP at the protein level (Fig. 6C). To understand the effect of FL3 in these U87-MG cells, a SA-beta Galactosidase assay was realized. In these conditions, most of U87-MG cells treated with FL3 were positively stained in blue in both normoxia and hypoxia (Fig. 6D, 6E). Moreover, mRNA expression levels of cell cycle regulators p16, p19 and p21 were increased in CoCl_2_ + FL3 treated cells, but not when cells were treated only with CoCl_2_ (Fig. 6F). Taken all together, these results clearly show that FL3 induced senescence in U87-MG cells cultured as well under hypoxia as under normoxia.

### FL3 with TMZ combination induces senescence in TMZ resistant GBM cells

**Figure 7:**
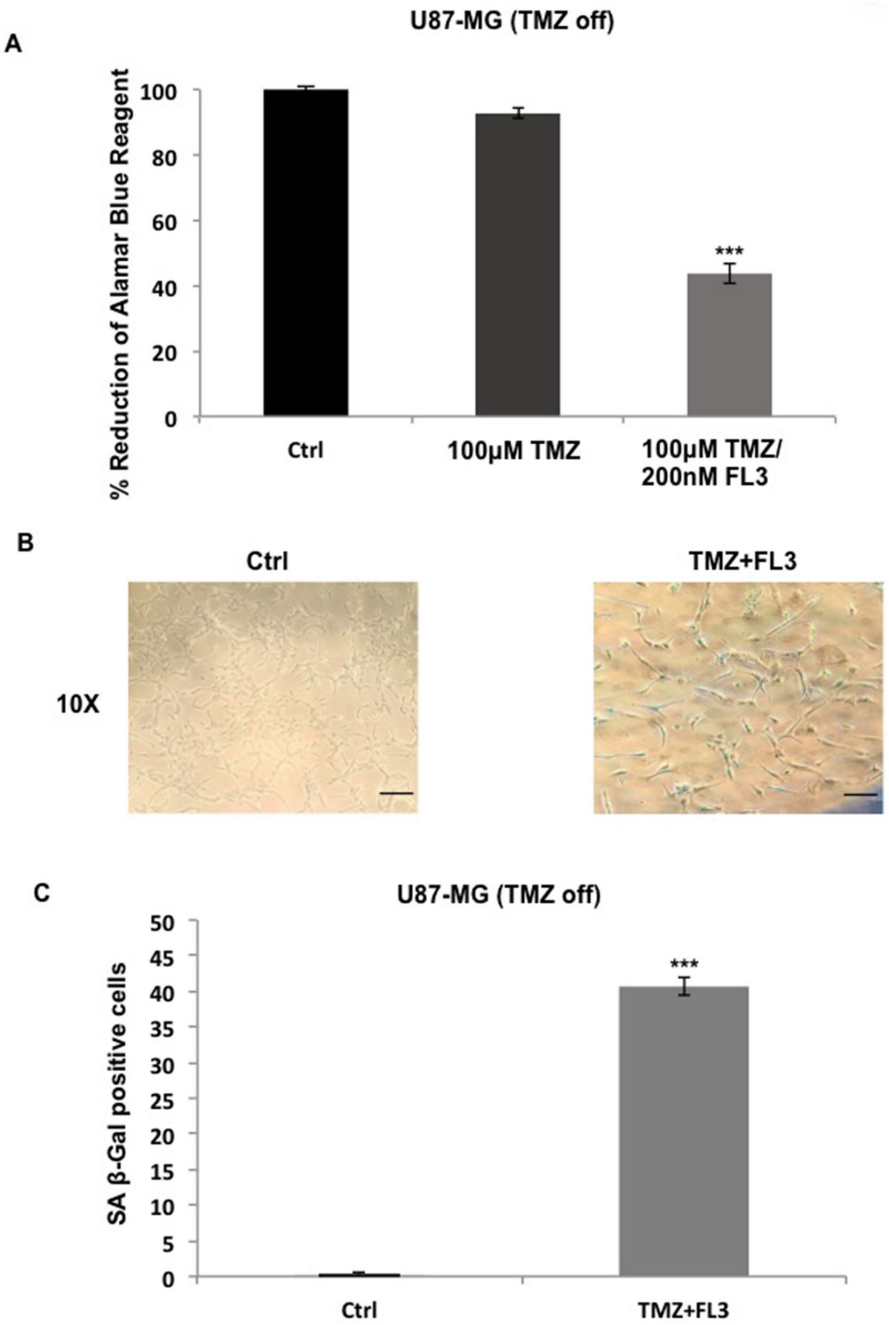
TMZ combination with FL3 induced senescence in U87-MG TMZ resistant cells. (**A**) Analysis of cell viability with the AlamarBlue assay in U87-MG (TMZ off) control cells, treated cells with 100 μM of TMZ and treated cells with 100 μM of TMZ + 200 nM of FL3. Quantification mean ± SEM from 3 independent experiments, ***P < 0.001 from control. (**B**) Representative senescence-associated β-galactosidase (SA β-gal) staining (blue) of U87-MG (TMZ off) control cells and treated cells with 100 μM of TMZ + 200 nM of FL3, 6 days after the FL3 treatment. Scale bar represents 100 μm. (**C**) Quantification of SA β-gal positive cells of U87-MG (TMZ off) control cells and treated cells 100 μM of TMZ + 200 nM of FL3. 50 cells were analysed for each condition. Results for mean ± SEM from 3 independent experiments, ***P < 0.001 from control.

In order to test the combination of FL3 molecule with TMZ treatment, we used a TMZ resistant GBM cell line: U87-MG (TMZ off) cells in the next experiment. We saw that FL3 inhibited the metabolic activity of U87-MG (TMZ off) cells (around 45%) compared to the control in contrast with TMZ treatment alone (Fig. 7A). On the other hand, U87-MG (TMZ off) cells were shown to be positive for the SA β-Gal assay 6 days post- FL3 treatment (Fig. 7B, C). Therefore, the use of FL3 molecule in combination with TMZ drug could be used as a treatment to induce senescence in TMZ resistant GBM cells.

### Human brain astrocytes are resistant to FL3

**Figure 8:**
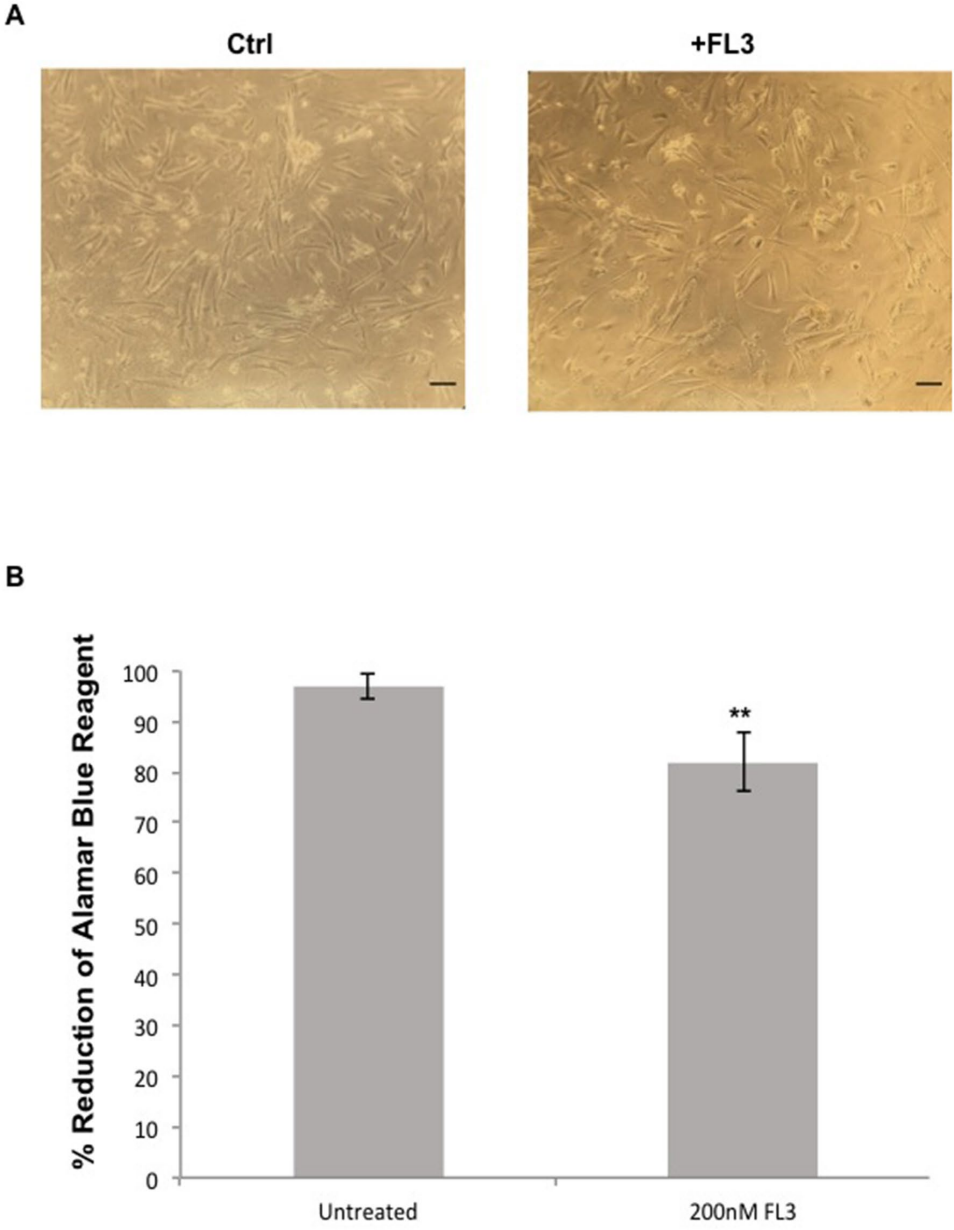
Effect of FL3 on Human brain astrocytes. (**A**) Representative images of control and treated human brain astrocytes with 200 nM FL3. Scale bar represents 100 μm. (**B**) Analysis of cell viability with AlamarBlue assay in human brain astrocytes without treatment and after treatment with 200 nM FL3 respectively. Quantification of mean ± SEM from 3 independent experiments, **P < 0.01 from control.

Since it has been described that FL3 is not toxic in non-cancer cells, we examined the effect of this molecule on normal human brain astrocytes in order to compare the results we obtained from treated U87-MG glioblastoma cells. When we incubated human brain astrocytes with 200 nM of FL3 for 48 h and by a simple visualization with microscope (Fig. 8A), we did not observe any remarkable difference concerning the number of cells between the control and treated cells. In addition to that and using the Alamar Blue assay, the results showed a slight decrease in the metabolic activity of treated cells (around 20%) with FL3 for 48 h, as compared to the control (Fig. 8B). In U87MG cells, same concentration of FL3 (200 nM) and time point of treatment (48 h), decreased the metabolic activity at about 70%. These results clearly showed that human brain astrocytes are considerably more resistant to the FL3 treatment than glioblastoma cells.

## Discussion

In this study, we evaluated the effect of the synthetic derivative of flavagline FL3 on different glioblastoma cell lines and compared normoxia to hypoxia conditions. FL3 is known to have anticancer effects and has been studied in several types of cancer cells but none of these studies was done on brain tumor cells. Here, we showed that FL3 decreases cell metabolic activity and proliferation by provoking cell cycle arrest at G2 phase in these cells (Fig. 1). We showed that FL3 provoked p38 phosphorylation and decreased Cyclin D protein expression as it was reported by Basmadjian et al.^4^. It has also been described that some flavagline derivatives could lead to cell cycle arrest at G0/G1 or G2/M phase in different cancer cell lines such as breast, colon, and prostate cancer cells^27–29^. Moreover, in the literature, these molecules caused apoptosis in cancer cells cultured in 2D models^27,28,30^. In our study, FL3 did not lead to apoptosis in U87-MG cancer cells (Fig. 2), in line with the results of Yuan et al*.* in bladder cancer cells. They found that FL3 induced cell cycle arrest at G2/M phase, without leading to apoptosis, supporting the fact that FL3 induces cell death in a tumor type-dependent way^31^. That is why it is an interesting perspective to see if the PHB is also implicated in the cell cycle arrest in glioblastoma cells, just like in bladder cancer cells. Indeed, in our study, classical flow cytometry analysis showed approximately the same number of apoptotic cells in treated conditions (with FL3) as compared to the control condition (without FL3), corresponding to the basal number of apoptotic cells. Moreover, PARP polymerase known to be involved in DNA repair, helping cell survival, is normally cleaved by caspase-3 when apoptosis occurs^32^, which was not the case in U87-MG glioblastoma cells treated with FL3. The Live/Dead assay results showed less cells when treated with FL3 compared with the control, indicating an inhibition of cell proliferation, but no dead cells were detected (Fig. 2B).

Cellular senescence takes place both in culture and in vivo as a response to excessive extracellular or intracellular stress. In cancer cells, cellular senescence can act as a cell-intrinsic mechanism to suppress tumor growth, by blocking the proliferation of these cells through the activation of p53, p16, p19, p21 tumor suppressor genes^33,34^. It can also act in an extrinsic manner through the development of the senescence-associated secretory phenotype (SASP). Senescent cells secrete cytokines, miRNA, pro-inflammatory mediators, interleukins, chemokines, extracellular proteases, and growth factors that can affect surrounding cells. Chemokines like CXCL1, CXCL2, CXCL5 are known to be upregulated in senescent cells^35,36^. There are few publications concerning the pharmacologically-induced cellular senescence in glioblastoma cancer cells (U87-MG)^37^ and it has been shown that U87-MG cells treated with temozolomide (TMZ) presents a G2/M cell cycle arrest, with an increase in p21 gene expression^38,39^. Moreover, these TMZ-treated cells were positively marked with the SA-β-Gal staining. In our study, the results obtained from the SA-β-Gal staining, the increase of nucleus size of glioblastoma U87-MG cancer cells, the significant increase of p16, p19 and p21 gene expression and the appearance of the SASP (CXCL1, 2 and 5 upregulated) (Figs. 3, 5), demonstrate an FL3-dependent induction of senescence in glioblastoma cancer cells. Flavaglines were already known to provoke apoptosis in cancer cells^4^. A recent study showed that these molecules can induce early autophagy in melanoma cells^40^, but our results showed for the first time that the flavagline FL3 promotes a cellular senescence in U87-MG glioblastoma cells. In addition, we showed in our study that FL3 provoked the decrease of the metabolic activity of both treated U373-MG and LN443 cancer cells, and that FL3 remarkably induced cellular senescence inside U373 and LN443 treated cells (Fig. 4). Taken together, our results clearly prove that FL3 induces senescence in glioblastoma cancer cells. Interestingly, U373-MG cells express a p53 mutant protein but were pushed towards senescence by FL3 perhaps with less efficacy than in p53WT-expressing U87-MG and LN443 cells. Results suggest that FL3-induced senescence may be partly p53-independent.

Hypoxia plays a key role in tumor progression and metastasis promoting angiogenesis and invasiveness. The resistance of cancer cells to treatment is significantly induced by hypoxia, making it a prime obstacle in anticancer treatments. For example, radiotherapy leads to Reactive Oxygen Species (ROS) production inducing DNA damage under normoxia, but this ROS production is disturbed under hypoxia^41,42^. Chemotherapy faces the same challenge of cancer cell resistance. Due to the abnormal vascularization seen in tumors and the formation of the hypoxic core in the centre of these tumors, chemotherapeutic agents do not reach well this hypoxic region. Moreover, hypoxic cells develop a resistance against chemotherapeutic agents by the overexpression of genes involved in apoptosis, cell proliferation and drug efflux^42,43^. For these reasons, hypoxia remains one of the main barriers blocking an efficient treatment. Here, we chose to mimic hypoxic conditions using CoCl_2_ agent. The cobalt of CoCl_2_ agent replaces the iron core (Fe) in prolyhydroxylases making them unable to mark HIF1α thus preventing its degradation. CoCl_2_ can also inhibit the interaction between HIF1α and von Hippel Lindau (VHL) protein, which is also implicated in the degradation of HIF1α^44,45^. Accordingly, after CoCl_2_ treatment, we could see an accumulation of HIF1α at the protein level, but not at the RNA level in U87-MG cancer cells (Fig. 5). The accumulation of HIF1α due to a lack of oxygen or an inhibition of protein degradation is reported to lead to the activation of its downstream genes, such as VEGF, GLUT1 and GLUT3^45^. We observed a significant increase in the expression of these three target genes in CoCl_2_ treated cells, indicating an upregulation of HIF1 transcriptional activity. Mitochondria constantly change shape through cycles of fusion and fission in response to various microenvironmental stimuli such as hypoxia. It has been shown that under hypoxia, mitochondria undergo fission^46^ in order to maintain the cellular integrity by decreasing their respiratory activity and keeping ROS production at a physiological low level. Accordingly, the mitochondrial fission is a supplementary marker showing that cells are in hypoxic conditions.

When we treated U87-MG cells with 200 nM of FL3 for 48 h under hypoxia, we observed a decrease of the cell metabolic activity at about 60% compared to the control (Fig. 6). A similar decrease of metabolic activity was found under normoxia with the same conditions for FL3 concentration and time of treatment (Fig. 1). No cleaved-PARP was detected in CoCl_2_ + FL3 treated cells and cells were positive for the SA-β Gal staining (Fig. 6), meaning that FL3 did not induce apoptosis, but senescence also in hypoxia. Thus, although hypoxia is known to induce resistance to treatments, FL3 is similarly efficient in hypoxia and in normoxia. FL3 therapy may be an interesting way to treat glioblastoma which are highly hypoxic..

The presence of TMZ resistant GBM cells is a major cause of treatment failure, that is why testing the combination of TMZ with other therapeutic molecules would be important to improve treatment outcomes. Our results showed that FL3 is able to trigger a senescence phenotype even in cells resistant to TMZ. The combination of TMZ with FL3 may thus be an interesting way to eliminate both TMZ sensitive and resistant glioma cells. (Fig. 7).

On the clinical level, chemotherapy leads in most cases to intolerable side effects. For this purpose, the discovery and the development of innovative therapeutics must focus on finding a tumor-specific therapy, in order to protect normal cells from the harmful side effects of chemotherapy. In this study, we showed that normal human brain astrocytes were not impacted by FL3 (Fig. 8).

In summary, we showed for the first time that the Flavagline derivative FL3 can induce senescence in glioblastoma cancer cell lines in normoxic or hypoxic conditions. This active molecule could be associated with the standard GBM chemotherapy TMZ or with other therapies to overcome intrinsic or acquired resistance..

## Materials and methods

### Cell lines and culture conditions

Glioblastoma U87-MG cancer cells were purchased from ATCC (HTB-14), U373 (Uppsala) cells were obtained from ECACC (Sigma Aldrich) and LN443 cells were kindly provided by Pr. M Hegi (Lausanne, Switzerland). All these cells are grown in MEM medium (L0430-500, Dominique Dutscher) with 10 U.mL^-1^ penicillin, 100 µg mL^−1^ streptomycin, 250 U mL^−1^ fungizone, 1 mM Na-pyruvate, 2 mM glutamine and 10% FBS. Human brain astrocytes were purchased from Alphabioregen (HBMP202) and cultured in their adapted medium (AGPM-03, Alphabioregen). Cells were incubated at 37 °C in a humidified atmosphere with 5% CO_2_. Cells were cultured and then treated once they reached 70–80% of their confluency inside the plates.

#### Establishing TMZ resistant GBM cell line

U87-MG cells were treated with 50 µM Temozolomide during 2 months. After this period, TMZ was retrieved from the culture medium and cells U87-MG (TMZ off) were regularly checked for their resistance to the drug by the Incucyte technology.

### Treatment of cells

*CoCl*_*2*_* treatment* 20 × 10^4^ cells per well were seeded into 6 well plates and then treated for 24 h with different concentrations of CoCl_2_ (50–300 µM) that were prepared from a stock solution of CoCl_2_ (10 mM) (C8661, MERCK).

*FL3 treatment* A solution of FL3 in DMSO (10 mM) was graciously given from Dr. Laurent Désaubry (Laboratoire d’Innovation Thérapeutique, UMR 7200, Strasbourg, France). U87-MG cells were treated with FL3 at different concentrations (20–4000 nM) for different time points (24, 48 and 72 h). The highest concentration of DMSO, for treated and non-treated cells, never exceeded 0.002% (v/v) in order to avoid its side effects such as cell toxicity or cell differentiation. In some experiments, cells were kept in culture for 2 to 8 days post-FL3 treatment. Human brain astrocytes were treated with 200 nM of FL3 for 48 h.

*FL3 treatment with CoCl*_*2*_ Cells were first treated with 150 µM of CoCl_2_ for 24 h, and then washed with PBS 1X and treated with 200 nM of FL3 for additional 48 h. Cells were kept in culture for 2 to 8 days post-FL3 treatment.

*TMZ treatment with FL3* Cells were first treated with 100 µM of TMZ for 72 h (we used this TMZ concentration to confirm that the cells are resistant to TMZ), and then washed with PBS 1X and treated with 200 nM of FL3 for additional 48 h. Cells were kept in culture for 6 days post-FL3 treatment.

### Metabolic activity

U87-MG cells were seeded into each well of 24 well plates at 5 × 10^4^ cells per well, and then treated with different FL3 concentrations (0, 20, 200, 1000, 2000 and 4000 nM) for 24, 48, and 72 h. AlamarBlue (Invitrogen, Thermo Fisher Scientific, Waltham, MA, USA) was used to assess cell proliferation over time. The AlamarBlue test is a non-toxic, water-soluble, colorimetric redox indicator that changes color in response to the cellular metabolism. At each time point, the cells were washed with PBS 1X and then incubated with 10% AlamarBlue solution diluted in phenol free medium (DMEM medium, BE12-197F, LONZA) for 4 h at 37 °C in a humidified atmosphere of 5% CO_2_. Each condition was prepared in triplicate. After 4 h of incubation, 150 µL of incubation medium from each well were transferred to a 96-well plate and the resulting absorbance was measured with a spectrophotometer (Multiskan FC, Thermo Fisher Scientific, Ratastie, Finland), at 570 nm and 595 nm wavelength respectively. Results were shown as the percentage of reduction of AlamarBlue reagent and the percentage of living cells was calculated as the ratio between the OD value of each FL3-treated cell sample and the OD value of the control. The AlamarBlue assay was also done on treated human brain astrocytes with 200 nM of FL3 for 48 h. We did the same protocol of AlamarBlue on U87-MG (TMZ off), U373-MG and LN443 cells.

### Cell cycle analysis

U87-MG cells were seeded into 6 well plates at 20 × 10^4^ cells per well, and then treated with FL3 for 48 h. Afterwards, cells were trypsinized and centrifuged to have a unique pellet. The cell pellet is washed with PBS 1X and directly fixed with ethanol 70% for 30 min at 4 °C. The cell pellet was washed with PBS 1X, centrifuged at 850 g, and then treated with 100 µg/ml of ribonuclease (Sigma Aldrich), to finally stain it with 200 µl of Propidium Iodide (PI) (*life technologies,* Sigma Aldrich) at a concentration of 50 µg/ml (prepared from a 5 mg/ml stock solution). After 30 min of incubation at room temperature in the dark, cells were examined using a flow cytometer (MACS Quant, Miltenyi Biotec GmbH, Bergisch Gladbach R. F. A). A minimum of 20 × 10^3^ cells was acquired per sample and data were analyzed using ModFit LT V3.3 software (Verity Software House, Topsahm, Maine, USA, https://www.vsh.com/products/mflt/). The percentage of cells in G0/G1, S and G2/M was determined from DNA content histograms.

### Apoptosis analysis

Apoptosis was evaluated by measuring the externalization of phosphatidylserine with Annexin V, and nucleus labelling with propidium iodide (PI). In this context, cultured cells were treated with different concentrations of FL3 for 48 h. Apoptosis rates were assessed using flow cytometry (LSR 2 with Diva 6.2 Software, Becton Dickinson Biosciences, San Jose California, USA, https://www.bdbiosciences.com/en-us/instruments/research-instruments/research-software/flow-cytometry-acquisition/facsdiva-software) using the Annexin V-FITC/PI Apoptosis kit (V13241, Thermo Fisher Scientific) according to the manufacturer’s recommendations.

### RNA extraction and analysis

Total RNAs were isolated from the U87-MG cell line. 1 µg of RNA was retro-transcribed using the iScript Reverse Transcription Supermix (Bio-Rad Laboratories, Hercules, CA, USA) according to the manufacturer’s instructions. Quantitative PCRs of retro-transcribed RNAs were performed and analyzed using the CFX Connect Real Time PCR Detection System (Bio-Rad, Mitry Mory, France). Amplification reactions had been performed using iTaq Universal SYBR Green Supermix (Bio-Rad, France). Actin was used as endogenous control (housekeeping gene) in all cDNA samples (See Table 1 for primers sequences). The mRNA expression levels were calculated using the comparative Ct method (2^−ΔΔCt^) and normalized to the housekeeping gene. All RT-qPCR assays were performed in triplicate and results were represented by the mean values.

**Table 1:** Sequence of primers used for Q-PCR.

Genes	Forward primer	Reverse primer
Actin	5′-GATGAGATTGGCATGGCTTT-3′	5′-CACCTTCACCGTTCCAGTTT-3′
GLUT1	5′-CTGAAGTCGCACAGTGAATA-3′	5′-TGGGTGGAGTTAATGGAGTA-3′
GLUT3	5′-GACCCAGAGATGCTGTAATGGT-3′	5′-TGGCAAATATCAGAGCTGGGG-3′
HIF1α	5′-CTGCCACCACTGATGAATTA-3′	5′-GTATGTGGGTAGGAGATGGA-3′
VEGF	5′-CCTGGTGGACATCTTCCAGGAGTACC-3′	5′-GAAGCTCATCTCTCCTATGTGCTGGC-3′
p16	5′ CTTCGGCTGACTGGCTGG 3′	5′ TCATCATGACCTGGATCGGC 3′
p19	5′ CGCTGCAGGTCATGATGTTT 3′	5′ GGGTGTCCAGGAATCCAGTG 3′
p21	5′ TGCCGAAGTCAGTTCCTTGT 3′	5′ GTTCTGACATGGCGCCTCC 3′
CSF1	5′ TCCAGCCAAGATGTGGTGAC 3′	5′ TCAGAGTCCTCCCAGGTCAA 3′
CXCL1	5′ CCGAAGTCATAGCCACACTCA 3′	5′ TTCTTAACTATGGGGGATGCAG 3′
CXCL2	5′ GAAAGCTTGTCTCAACCCCG 3′	5′ TGGTCAGTTGGATTTGCCATTTT 3′
CXCL5	5′ CAGACCACGCAAGGAGTTCA 3′	5′ TCTTCAGGGAGGCTACCACT 3′
TNFα	5′ CTTGCGCAATGCCACCCA 3′	5′ TTCATCTTCAGCAGCCGGTC 3′

### Preparation and analysis of protein extracts

Cells were lysed in ice-cold radioimmunoprecipitation assay buffer (50 mM Tris, pH 7.5, 150 mM NaCl, 0.5% sodium deoxycholate, 0.1% SDS, 1% Triton, supplemented with protease inhibitor cocktail (Sigma-Aldrich). Protein extracts (50 µg) were electrophoresed on 10–15% SDS–polyacrylamide gels and electroblotted to Hybond nitrocellulose membranes (Invitrogen) using iBlot transfer (Invitrogen). Proteins were detected using primary antibodies directed against cleaved PARP 552596 (BD Biosciences), HIF1α (ab17983, Abcam, 1:1000), cyclin-D1 (sc-8396, Santa Cruz, 1:1000), phospho-p38 (9215, Cell Signaling, 1:1000), β-actin (66009-10-Ig, Proteintech, 1:5000), GAPDH (60,004–10-Ig, Proteintech, 1:5000), β-tubulin (10094-1-AP, Proteintech, 1:5000). Membranes were probed with HRP-conjugated mouse (A90-147P, Bethyl Laboratories) and rabbit (sc-2004; Santa Cruz) secondary antibodies, and were detected using ECL chemiluminescence substrate solution (SuperSignal West Pico Plus, Thermo Scientific, USA). Autoradiographic signals were captured on an iBright Imaging System (Invitrogen) and analyzed by the NIH’s Image J software (Supplementary Fig. 1).

### SA-β gal staining

U87-MG cells were seeded into 24 well plates at 5 × 10^4^ cells per well, and then treated with FL3 for 48 h. Afterwards, the treatment was stopped, cells were washed with PBS, refilled with new fresh medium and incubated for 8 days. Medium was changed every two days. At each time point (2, 4, 6 and 8 days), an aliquot of the cells was fixed and stained with the senescence β-galactosidase staining kit #9860 (Cell Signaling), and then kept in a dry incubator (without CO_2_) at 37 °C overnight. Cells were checked under an inverted microscope (Nikon Eclipse TS100, Nikon) for blue color detection and photos were taken using the NIS element software (Nikon). We did the same protocol of SA-β Gal staining on U87-MG (TMZ off), U373-MG and LN443 cells.

### Live/dead assay

U87-MG cells were seeded onto coverslips in 24 well plates at 5 × 10^4^ cells per well, and then treated with 200 nM of FL3 for 48 h. After 48 h, the treatment was stopped, and the wells were washed with PBS. The cells were trypsinized and then centrifuged to have a unique pellet. The cell pellet was resuspended in the live/dead assay dye solution for 10 min (Live/dead cell assay kit, Abcam, ab115347), and then visualized under an epifluorescence microscope (Leica DM4000 B).

### Immunofluorescence

U87-MG cells were seeded onto coverslips in 24 well plates at 5 × 10^y^ cells per well, and then treated with CoCl_2_ for 24 h. After 24 h, the media was changed, and the wells were washed with PBS. Later, the cells were fixed with PFA 4% for 10 min, rinsed with PBS, permeabilized and saturated with PBS—0.2% Triton—3% BSA solution for 30 min. Then, the cells were incubated with the Cytochrome C 556432 (BD Biosciences) primary antibody diluted at 1/300 for 45 min. After three washes with PBS, cells were incubated for 1 h with anti-mouse secondary antibody (diluted at 1/200) conjugated to Alexa Fluor 594 (A21203, Thermo Fisher Scientific). The cells were washed with PBS and incubated for 5 min with Hoescht 33342 (H3570, Invitorgen, Thermo Fisher Scientific) diluted at 1/10,000 in PBS in order to stain the nucleus in blue. Cells were visualized under a fluorescence microscope (Nikon Eclipse 80i, Nikon).

### Statistical analysis

Statistical analyses are performed using *t*-test. All data are presented as mean ± SEM from three independent experiments. *P < 0.05; **P < 0.01; ***P < 0.001.

## Supplementary information

Supplementary Figure.
