# Supplementary figures and images for: Flavagline synthetic derivative induces senescence in glioblastoma cancer cells without being toxic to healthy astrocytes
Source: Sci Rep. 2020 Aug 13;10:13750. doi: 10.1038/s41598-020-70820-6 (PMC7426813; doi:10.1038/s41598-020-70820-6)

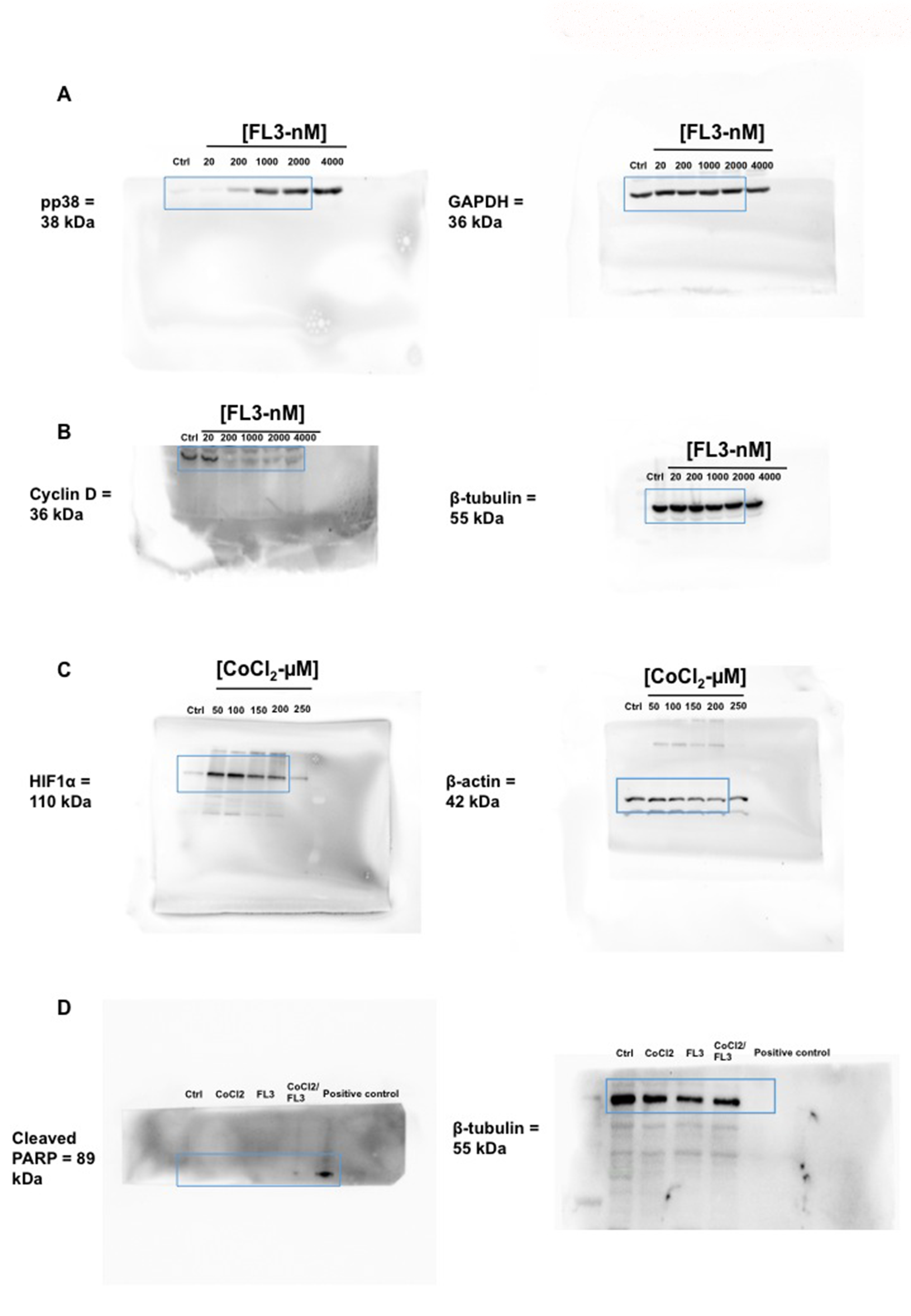

Supplement: Supplementary file 1 — Supplementary Figure. [file 41598_2020_70820_MOESM1_ESM.tif]
